# Sycosis*Read before the Bœnninghausen Club, March 14th, 1895.

**Published:** 1895-05

**Authors:** Frederick S. Keith

**Affiliations:** Waltham, Mass.


### SYCOSIS.*

FREDERICK S. KEITH, M. D., WALTHAM, MASS.

By sycosis is meant a constitutional and contagious disease, whose primary manifestation is a gonorrhœa.

Before Hahnemann's time gonorrhœa was universally regarded as a purely local affection and treated accordingly. But it was observed that in some cases excrescences accompanied or soon followed the appearance of the discharge, and, an explanation being necessary, their growth was attributed to some special irritant property of the discharge (just what that was does not appear), or they were said to be a syphilitic manifestation. At that time the syphilitic origin was the more common theory, consequently the economy was assailed with massive doses of mercurials, of course without benefit. The warts were at the same time treated with the ligature, knife, or cautery.

Hahnemann evidently considered the primary manifestation of sycosis to be the fig-wart, for he says, in the *Chronic Diseases*, after referring to the violent external treatment of the excrescences: "The natural and immediate consequence of such violent treatment was that the excrescences generally came out again, and were again subjected to painful and cruel treatment. In case, however, they did not re-appear in their original form, they broke forth in the shape of more disagreeable and more dangerous secondary ailments; for neither the violent removal of the external embodiment or vicarious symptom of sycosis,

---

* Read before the Bœnninghausen Club, March 14th, 1895.

nor the internal administration of Mercury, which is not homogeneous to the miasm of sycosis, had the least influence in diminishing the intensity of this miasm and preventing it from affecting the whole organism."

The same idea that the fig-wart was the primary symptom seems also to be conveyed in the following: "The excrescences first appear upon those parts of the body several days or weeks after the infection by the act of coition has taken place. They are accompanied" (note the word *accompanied*) "with a sort of gonorrhœa from the urethra, are sometimes dry and in the form of warts, but more frequently soft, spongy, emitting a fetid fluid, *sui generis*, of a sweetish taste (almost resembling that of herring-pickle), bleeding readily and having the form of a coxcomb or a cauliflower."

Note also *Organon*, § 205 and foot-note: "Homœopathic practice never requires us to single out some primary or secondary symptom resulting from chronic miasms nor to resort to external local remedies either dynamic or mechanical. But wherever one of these symptoms appears Homœopathy cures the great fundamental miasm, together with which its primary as well as its secondary symptoms vanish simultaneously. But as the homœopathic physician will generally find the primary symptoms * to have been suppressed by local treatment of allopathic practitioners, it will be his duty to accomplish what his predecessors neglected to do. He will, therefore, give his attention more particularly to the secondary symptoms which result from the development of an inner miasm. . . ."

Hahnemann clearly saw that he had to deal with two classes of urethral discharges, for in referring to the discharge accompanying the warts he says: "In this kind of gonorrhœa the fluid which comes out of the urethra looks like thick pus." While in another place we find: "The miasm of the common clap seems to affect the urinary organs only locally; it does not pervade the entire system."

In other words, it seems from the accumulated evidence since

---

* Itch, eruption, chancre, bubo, condyloid excrescences.

his time that Hahnemann assigned to the venereal wart too high a place in the sycotic miasm, and gave too little weight to the suppression of the gonorrhœal discharge.

The practice of over half a century by Hahnemann's faithful followers only serves to emphasize more and more strongly the truths of the master, and as he left psora, so with little in addition it stands to-day. But of sycosis and syphilis we have merely a hint, a foundation, as it were, from which we must build. And the wonder is not that he did not go on with sycosis, but how one lifetime could suffice to accomplish all the immortal Hahnemann left us.

What he does not insist on in so many words is the ill effects of suppressing the discharge, regardless of the presence or absence of the warts. It seems that this point should be very strongly insisted upon, for many undoubted sycotic cases are seen where there have never been any warts or excrescences about the genitals or any other part of the body.

Our literature is very scant on sycosis, and this seems remarkable and deplorable when we consider the vast importance it bears to the welfare of this and following generations. The subject has not been given a tenth part the consideration it deserves; and can we hope for better results until cases are observed with diligence and care, and careful records of the symptoms and results of treatment are kept?

No hasty prescriber, anxious to cover each varying image of the disease, no careless routinist will ever investigate sycosis, a miasm just as deep as either syphilis or psora, and often far more insidious and difficult of management.

An article, "Thuja as an Intercurrent Remedy," by Bœnninghausen, in the *American Homœopathic Review*, Vol. III, is full of interest. He speaks of the value of Sulph. as an intercurrent in many cases of acute and chronic disease in which a remedy, though accurately selected and strictly homœopathic, does not act. Continuing, the use of Merc. as an intercurrent in secondary syphilis is referred to.

I quote from the paper the following: "On the other hand, so far as I know, a similar use of our great antisycotic remedy,

Thuja, has not hitherto been customary, and it may not be amiss to call attention to it. If it is true that

"1. Variola and cow-pox belong naturally to the order of sycosis; that

"2. This miasmatic poison has received a prodigious propagation through the customary process of vaccination, and that, finally,

"3. Many chronic affections of the worst character prove intractable under our best remedies, and show no signs of improvement, until recourse is had to a remedy which has the power of acting favorably upon diseases of a sycotic character—if these things be true, then the circumstance is of sufficient importance to warrant a few words:

"1. If I am the first to utter the conjecture that condyloma and variola (or vaccinia) belong to one and the same disease, I base my hypothesis on the fact that both affections, so long as they present themselves without complication, find their surest and most complete cure in one and the same remedy, viz., in the juice of the Thuja Occidentalis, and in no other. . . .

"This much is certain, that no one of all our numerous remedies possesses so great and uniform a curative influence over small-pox, and that therefore the above proposition is not without justification, even though these facts be no incontrovertible demonstration of its truth."

To sum up the proposition it is: Thuja is prominent as an antisycotic, Thuja is often indicated in small-pox, therefore small-pox is likely to be a form of sycosis. This seems far from conclusive. Sycosis is one of Hahnemann's three chronic miasms, and like the other two pursues its relentless, progressive, ever downward course. Small-pox is essentially and entirely different in its nature. It is an acute disease, an acute miasm terminating either in recovery or death. Here is the difference in their nature: In the acute miasm the alternative, in the chronic no alternative, but final dissolution inevitable, unless homœopathically treated.

Puls. is often indicated in measles; Puls. is an antisycotic of no mean rank. Shall we then conclude that measles and sycosis are one, or, more accurately, that measles is a sycotic manifestation?

Bœnninghausen further says: "2. The general distribution of this sycotic poison, which was indicated even by Hahnemann as one of the miasmatic poisons out of which chronic diseases spring, through the practice of vaccination, needs no further discussion if it be conceded that our first proposition is correct. We need only call attention to the thousand-fold experience that many children, who were previously in perfect health, begin, not long after vaccination, to become ailing, and, what is most remarkable, they become ill of such varieties of chronic diseases as require most frequently such remedies as are related to Thuja and such as may even be called in play in treating real condyloma."

Then follows a list of remedies useful in condylomata and sycosis. It is, however, true that in the list given, many of the remedies are fully as important antipsorics as antisycotics. And on that argument why should we not attribute the conditions brought about by vaccination as much to psora as to sycosis?

The third proposition stating the value of Thuja as an intercurrent has, however, a practical bearing on the treatment of this miasm, for no one can call in question the marvelous power of observation possessed by this great prescriber.

The "Anamnesis of Sycosis," *American Homœopathic Review*, by Bœnninghausen, should also be referred to.

This briefly is a list of the special symptoms of Thuja not found under either Sulph. or Merc. It therefore covers only as much of sycosis as is found in that miasm in common with Thuja. It is no more complete in detail than would a study of psora be, based on the pathogenesis of Sulph. or syphilis from that of Merc.

The practical question for us as physicians is how to recognize sycosis clinically in all its branches. Take any result of disease and how shall we answer the question, is this the result of psora, syphilis, or sycosis? Here we come to something that no abstract reasoning can settle for us, only strict adherence to principle and a careful application of the homœopathic law will help us in the recognition and cure of this dread disease.

There are two ways of studying sycosis, as with syphilis or psora: 1. Careful observation of the course of the disease from the beginning. 2. Observation of cases under treatment.

1. This method can have but a limited value when the wholesale and promiscuous drugging is taken into account. A case uncomplicated by drugs is rare—yes, almost unknown. So that we cannot judge how long the discharge would remain of itself, thus preventing deeper ravages, nor what the future course would be after its cessation.

2. The observation of cases under homœopathic treatment affords us sure indications for placing the conditions in the right group. We *know* that symptoms (diseases) get well from above downward, from within outward, and in an inverse order of their occurrence. There is no other way. If after a prescription for headache, backache, a tumor or what not, these conditions disappear and a urethral discharge takes their place, what can be more certain than the class to which they belong? That which comes after an orderly prescription shows the nature of that which preceded.

Let us start with the primary contagion and follow the disease in some of its more common expressions.

In the first place, as to the discharge, is it possible to tell in the beginning whether we have to deal with a case that has in it the miasm of sycosis, with its long train of symptoms if suppressed, or a case of simple urethritis? In many cases I believe it absolutely impossible. Hahnemann tells us that in the specific cases "the fluid which comes out of the urethra looks like pus," and that "micturition is not very painful, but the penis feels hard and swollen. Upon its back it is sometimes covered with glandular tubercles, and it is very painful to the touch." The "bug-hunters" look for the gonococcus. But, clinically, when the easy access to drug-stores and the "sure-cure-in-a-week" remedies are considered, and the effect these have on a case before it comes under observation, I should hesitate before asserting that this case is sycotic, that case is not.

Secondly, can we predict from the intensity of the early symptoms and the duration of the discharge the severity of what is to come? I believe not.

A high grade of inflammatory conditions has been followed after suppressive treatment by no sycotic manifestations, and on the other hand a mild and apparently insignificant primary stage has furnished the foundation for life-long suffering.

The duration of the discharge affords no clue, for here, as in local treatment of other affections, various factors come into account. The discharge will be much easier suppressed in some constitutions than in others, and those discharges which are most easily suppressed bring in their train the worst results. The ordinary gleet lingering for month after month is not indicative of sycosis, but is due to psora and analogous to a catarrh from any other mucous membrane.

What can be generalized from this? Simply that each case, even the mildest, may hold within it the miasm of sycosis, and must be treated with the possibility of all the secondary manifestations in view, from the fact that we cannot in the beginning predict its future course. And, considering this, every effort must be made to avoid suppression of the discharge.

While this paper is not concerned with the treatment of gonorrhœa *per se*, it may be worth while to ask what constitutes a suppression? I think here it is too apt to be considered synonymous with allopathy and syringes. This is undoubtedly wrong, and I believe to-day that almost as much harm may be done by the low potencies or frequent repetition of the dose, or both, as is usually the case, as by injections. When you read of the "cure" of gonorrhœa in a few days by the low potencies or frequent dose, you can put that case down as a suppression, a substitution of the remedy for the disease. There can be no question of the fact that symptoms may be suppressed by frequent doses of even the highest potencies without a cure of the disease. What the treatment of gonorrhœa needs by the profession is that sort of prescribing in which a remedy will be carefully selected and allowed to complete its action before repetition or change. Let us not be in a hurry to deprive the patient of his only salvation—the discharge.

During the ordinary local suppressive treatment of gonorrhœa, inflammations of adjacent structures are set up and we have orchitis, prostatitis, cystitis, and even nephritis. In the sycotic form, more tendency to remain and become chronic would be expected—chronic induration of the testis and spermatic cord. In the female, the immediate results of suppression may be inflammation of the uterus, tubes, ovaries, and the starting-point of all the great host of gynæcological affections which, together with the bad treatment they receive, go far to make the existence of such a patient a living death. Think of the long list and add to it the so-called hysterical, reflex, sympathetic, nervous symptoms (the classification doesn't matter—no two of the authorities agree), and does it not seem incredible that physicians can go on blindly following the same routine of local suppressive treatment? In both sexes the disease is denied its natural outlet, and fixes itself on the nearest organ.

The earliest of the secondary manifestations, though they are also seen at other times, is the throwing out of warts and excrescences about the genitals. Here we see the effort to keep an external condition and protect the interior. Partly suppress the discharge, and at once, or after several days or weeks, the well-known venereal wart makes its appearance. Nothing is to be added to Hahnemann's description of them, previously quoted. In the strong, vigorous constitution, with its outward (*i. e.*, healthful) tendency marked, we should expect the venereal wart rather than in the weakly, run-down individual. Not by any means all have the vitality to throw out warts after suppression of the discharge; in fact, but a small proportion of cases have them. In poor constitutions, the disease is transferred toward the vital centres—the inner man is assailed, instead of the outer. If the warts appear, the next effort of the gross material thinker, and they are legion, is to rid the organism of this expression of the miasm of sycosis. They do not understand that, next to the discharge, these out-growths are the most harmless manifestation of the disease, because most external. What was true in Hahnemann's time is no less the fact to-day, that the warts are cut and tied and burned, and the larger the mass of excrescences, the bigger and more puffed-up the operator who removes them feels. Instead of being thankful to have a patient with the vitality to produce a large crop of warts, they blindly and ignorantly refuse the internal disease its outward expression.

Their removal is followed by re-appearance, or else more serious, more internal affections take their place.

Gonorrhœal rheumatism ("gonorrhœal synovitis," some of our allopathic brethren prefer to call it—they have such a liking for naming diseases) has been for years a bug-bear to the profession, and the theories and speculations to account for it, and, at the same time, maintain gonorrhœa a local affection, are numberless. The whole mass of theory may be dismissed without discussion. To us, as homœopaths, it is worthless. With our law of cure, we can at once assign it to its proper place—sycosis. This is one of the most common and best understood of the sycotic conditions, and of itself (leaving all other results aside) should be convincing and perfect proof of the existence of this miasm. Such, however, is not the case. The trouble may begin shortly after suppression of the discharge or removal of the warts, or not for some time. Striking in the gonorrhœal rheumatism is the day aggravation, and it may be said in passing that the same is prominent in the whole miasm. All joints may be affected, but the knee and ankle are most common. The suffering is intense, with great excitement and restlessness. He is in constant motion, is worse before a storm, relieved by the storm. A correct prescription is followed by cure of the pains and reproduction of the discharge. In the absence of this, there is no tendency to recovery. The trouble stays in the knee, or more often goes down to the ankle or heel, and there it remains month after month. The joints become stiff, contraction of tendons may occur. The soles of the feet become sore and tender, walking being well-nigh or quite impossible.

But in the great majority of cases nothing so decisive as the gonorrhœal rheumatism develops. A sycotic individual may refer the beginning of his ill-health in an indefinite way to his attack of gonorrhœa, yet complain of nothing definite for years.

These later conditions of sycosis offer the greatest difficulty in their investigation, from the great care necessary in the prescription, and the long period during which they must be watched to understand their backward course under treatment. Not all cases have the vitality to reproduce the discharge after the deep ravages of the miasm. It may take two or three years of the most careful selection of complementary remedies before the individual is sufficiently built up to throw out the discharge. Not all patients will remain under observation so long. The results of disease in organs may be so far advanced as to render a cure impossible. Yet with all these and many other difficulties, a large number of diseases have been investigated, and in many instances verified by the appearance of fig-warts, or the discharge, if there was one.

And this brings us, before entering into a general description of the miasm and the organs and tissues affected, to a consideration of the contagiousness of sycosis. Is it possible to have sycosis without the individual having had a gonorrhœal discharge? It unquestionably is. The disease can be communicated years after the discharge has been suppressed, as from a husband to the wife. And she will take up the disease as she receives it—in the secondary form. Here, as for the contraction of any disease, the element of susceptibility comes in, and the closer their relations, the closer they enter each other's life, the more the chance of contagion. The fact of contagion in the secondary stage will not be accepted by all, some preferring to argue that we have a small amount of gleety discharge remaining, by which the disease is communicated. In answer to this it may be stated at once that a gleety discharge lingering for months is generally psoric and not sycotic. And as a matter of fact it is not the individual having a gleet, however slight, who does the harm, but one who was absolutely "cured" (of the discharge) in two or three weeks.

Whether the miasm is viewed in the male as following the suppression of a gonorrhœa, or in the female, taken either primarily or in the secondary form, certain features are common to both and of value in its recognition.

Very characteristic is the pallid, anæmic appearance. The countenance is waxy, gray, shiny, transparent. It has a sickly look. The blood is thin and watery, and often a hemorrhagic tendency is brought about. A disposition to build is seen on the surface, and warts and excrescences appear about the genitals, anus, and even lips, ears, and nose. Crusts that crack and bleed grow on the skin. Proliferation of epithelial structures occurs. Epithelioma will probably be found directly traceable to sycosis. Large seed-warts, sensitive and easily cracking and bleeding, are many of them sycotic.

Any portion or organ of the body may take on the results of this miasm. Sycosis is not limited to fig-warts, gonorrhœal rheumatism, stricture, but it seeks man's very interiors, dethroning even the will and the understanding.

Mental symptoms of all degrees are seen. Irritability, anxiety, hopelessness, despair, melancholia, doubts of recovery, doubts of salvation. Weakness of the memory. Inability to apply the mind, and a host of others, even various forms of insanity.

Study carefully the provings of Medorrhinum and their verifications, and we cannot but be impressed by the profound and general effect the poison has on the economy, and the mind takes its full share of the universal disturbance and suffering. All the organs seem affected, and there is no question that, as further provings of Medorrhinum are made and as experience broadens in the future, many of the results of disease now attributed to psora will find their cause in sycosis or a complication of the two miasms.

The lungs are affected by sycosis. Asthma, wheezing, rattling breathing, dyspnœa. Worse in damp weather. Phthisis pituitosa, catarrhal phthisis, the lungs break down. When expectoration is copious he is somewhat relieved, but suffers more as the expectoration is scantier.

Catarrh of the nose after suppressed gonorrhœa, the profuse discharge is an outlet and saves him from deeper troubles. He is a great sufferer if the discharge lessens. It may be green, yellow, white, or milky often.

Heart disease has been traced to sycosis.

Evidence seems to warrant the placing of some cases of Bright's disease in this group. Possibly diabetes also.

The stomach, liver, and spleen have been found affected.

A very prominent sphere of action is the genital system of both sexes. In the male are found chronic induration of the testicle and spermatic cord, impotence with its accompanying mental symptoms. In the female a complete list of the diseases would undoubtedly comprise almost all the affections known to the gynæcologist.

Schrœder, a prominent "old school" authority in Ziemssen's *Cyclopædia* says: "Nœgerrath, of New York, holds that gonorrhœa in men is incurable; that when it is apparently healed it has only become latent, and in case of marriage it is invariably communicated to the wife. The latter contracts an inflammation of the mucous membrane which extends from the entrance of the vagina to the ovaries. Nœgerrath follows out this view very closely. He meets the objection that considering the commonness of gonorrhœa (80 % according to him and Ricord) all the wives should be diseased, with the reply, 'And they are all diseased. It has come to such a pass that young ladies are afraid to get married because they know that all of their married acquaintances were made ill directly and never again recovered.' Gonorrhœa in women, according to him, occurs in the form of an acute perimetritis (sometimes puerperal), a recurrent perimetritis, or an ovaritis. But the catarrh of the Fallopian tubes plays the most important part in the affection. A sudden escape of but a few drops of the inflammatory secretion (occasioned by a contraction of the tubes) may give rise to any of the various forms of perimetritis, including even the rapidly fatal acute peritonitis. Sterility, also, is very commonly due to a latent gonorrhœa and in the event of conception, abortion, premature delivery, and perimetritis during gestation are exceedingly apt to follow. Nœgerrath's assertions are undoubtedly extravagant, yet we are forced to admit that the chronic inflammatory condition of the genital organs—the endometritis, metritis, and perimetritis—are only too apt to be the result of gonorrhœal infection."

To Schrœder, as he says, these statements of Nœgerrath were "extravagant," yet every word of them is full of interest, and the profession takes a somewhat broader view to-day than at that time.

And yet they cling to the material theory, and talk as if the gonococcus somehow was carried up through the vagina, uterus, and tubes and finally lodged on the ovary, and there executed his nefarious designs. This theory of extension must be given up in many cases, as it cannot be reconciled with the facts. The troubles do not always take the order which extension of inflammation would suppose. In some cases the symptoms are not at first those of the genital organs, but often various indefinite symptoms seemingly not directly connected therewith. The importance of this consists in the conclusion that a woman may become sycotic after marriage with a sycotic husband, and not manifest the contagion by any leucorrhœa or other acute local signs of a gonorrhœal infection. No immediate results may be seen, and perhaps after years she may have a cyst or tumor of the ovary or its ligaments. The pathologist will see no relation, but will examine the cells and the fluid and enter into long discussions about embryonic tissues, etc., as if a thing can exist of itself without some prior cause—*i. e.*, be the cause and the effect in one.

Sycosis is a constitutional disease, and has a marked affinity for, a tendency to ultimate itself in the female upon the sexual system. As previously stated, the list of affections is a long one. Terrible dysmenorrhœa, most intense suffering is experienced. Inflammation, even ulceration of the os, cervix, and uterus are not rare. Inflammation of the Fallopian tubes, pyo-salpinx, hydro-salpinx, various diseases of the ovaries, malpositions of the uterus, symptoms of the bladder, have all been traced to this cause.

A very important feature, and it is Providential, so far as it saves the future generations, is sterility. Sycotic marriages are not fruitful. Commonly no children are born, or but one. We can conceive of cases where the woman has strong vitality, only slight susceptibility to the husband's nature, and great resistance to morbific influences. In such a couple marriage may be more productive. The general statement, however, is true that the number of births is greatly lessened. The percentage of abortions and premature births, however, is greatly increased, and the complications are rendered greater.

The hereditary form of sycosis offers a tremendous field for future study. In this stage of the disease we cannot have the characteristics of the miasm as clear and evident as in the secondary form. The disease is complicated with psora or syphilis, or both, and handed down as a unit, not with one group of symptoms or the other uppermost (as is often the case in the secondary stage, unless maltreated).

The infant has the appearance of a little old man, unhealthy aspect, fine, frizzy, hairy growth on the face. They often take on marasmus. Cholera infantum is frequent in the sycotic, Medorrhinum being the only remedy that will save the case. These children are unusually exposed to and affected by the diseases of childhood. Disorders of the mouth and stomach are frequent. The bones are soft; child is late learning to walk. Infants wheeze, often become asthmatic at puberty. Most of the asthmatic children are sycotic. Warts and excrescences are often seen about the genitals of children of both sexes. They may come without any treatment, and show the effort of nature to establish an outward expression. Children of strong vitality are the ones who have them. Women of good constitution, virgins, show this expression of sycosis.

The tertiary conditions are very hard to trace, and require great tact and knowledge for their investigation. It is probable that many young men and women, often the only child born to the parents, are treated, where sycosis is at the foundation of the trouble, and unsuspected. Man does not publish his misdeeds far and wide. He hides them, and from none more than his children.

The prognosis of sycosis should be referred to. The cure, where the trouble dates from a suppressed gonorrhœa in that patient, consists in the return of the discharge and its subsequent cure by the internal remedy. There is no other way.

Any case in which the discharge does not return cannot be considered a cure, no matter how much the symptoms are palliated and changed by remedies. Without a return there is no permanency. Where sycosis has been taken in the secondary form, as by a woman from her husband, the cure is the growth of fig-warts or cauliflower excrescences and their removal by the homœopathic remedy. Large masses grow from the cervix, sometimes almost occluding the vagina. In the hereditary subject, the progress toward cure is manifested by warts about the genitals. The anus may be almost surrounded by them. These, in their turn, are cured.

Thus, we see in sycosis, as in syphilis or psora, the outward course of symptoms in the progress to health. The returning discharge often lingers for months, the excrescences thrown out often prove most intractable. This should be expected and is according to law. Not until the internal disease is completely eradicated should these external embodiments disappear, and it is not to be expected that life-long miasms can be cured in a few weeks. Months and even years are required.

The treatment of sycosis, as of all other miasms, should be conducted exactly according to the teachings of the master, as given in *The Organon.* Here, as always, the totality of symptoms furnishes the only indications for a remedy. Let us not, however, consider its treatment an easy task, for in no class of cases is the arrival at the simillimum more difficult. Nowhere will the skill and patience of the prescriber be more tried than in the development and cure of this miasm in its multitudinous forms. Skill in selection of a remedy and its complements, patience in waiting for a remedy to complete its action, are imperative.

I believe sycosis to be not one bit less deep-seated and fatal than either syphilis or psora. A common saying among young men is that " Clap is no worse than a bad cold," a thing easily taken and easily cured ; some at that age seem almost to be proud of it. But, alas ! they know not the insidious but relentless nature of the destroyer.

